# High blood pressure and lost EDHF response in redox dead Cys42Ser PKGIα knock-in mouse

**DOI:** 10.1186/1471-2210-11-S1-O22

**Published:** 2011-08-01

**Authors:** Oleksandra Prysyazhna, Olena Rudyk, Philip Eaton

**Affiliations:** 1 Department of Cardiology, King’s College London, London, SE11 7EH, UK

## Background

We previously showed PKGIα forms an interprotein disulfide between its two subunits in response to oxidants such as hydrogen peroxide (H_2_O_2_). This activates PKGIα independently of the classical NO-cGMP pathway. The oxidative activation of PKGIα may contribute to the endothelium-derived hyperpolarising factor (EDHF) phenomenon, especially as oxidant species such as H_2_O_2_ have been implicated as this factor.

## Results

To investigate this further we generated a Cys42Ser PKGIα knock-in (KI) mouse line. Immunoblotting confirmed that tissues from KI mice express PKGIα at the same level as wild type (WT). However, when KI hearts were perfused in Langendorff mode and exposed to H_2_O_2_ (50μM, 10min) they did not form a disulfide dimer as anticipated, whereas WT hearts did. Similarly aorta and mesenteric vessels from WT, but not KI, mice formed disulfide in response to H_2_O_2_. To examine the functional contribution of PKGIα disulfide dimerisation to oxidant-induced vasodilation, we compared the response of isolated rings of thoracic aorta and second order mesenteric arteries from WT or KI mice to H_2_O_2_. WT or KI rings of aorta that were preconstricted with EC80 phenylephrine (1μM) and then serially exposed to increasing concentration of H_2_O_2_ (n=3, 10 rings per group). KI aortas were resistant to H_2_O_2_-induced relaxation, showing a ~40% deficit in their maximal response. Second-order mesenteric arteries were preconstricted with EC80 u46619 (0.5μM) and then relaxed by exposure to increasing concentrations of H_2_O_2_, observing a significant rightward shift (insensitivity) in the KI dose-response compared to WT. To assess whether PKGIα disulfide activation contributes to the EDHF phenomenon we compared WT and KI relaxations to acetylcholine chloride (ACh,1 μM) in vessels with or without inhibition of NO (L-NAME 300 μM, 30 min) and prostanoid (indomethacin 10 μM, 30 min) synthesis. The EDHF response was absent in aorta regardless of genotype. In contrast the EDHF response accounted for 30% of total ACh relaxation in WT mesenterics. KI mesenteric EDHF relaxation was absent and total ACh response was significantly attenuated. To assess the importance of these events in vivo we used blood pressure telemetry monitoring. Blood pressure (SAP, MAP and DAP) was significantly higher in KI mice than littermate WTs.

## Conclusion

PKGIα disulfide formation is a significant component of oxidant-induced vasodilation, consistent with this being a major component of the EDHF phenomenon. Furthermore, this mechanism operates basally to control blood pressure, as its genetic removal in the KI results in hypertension.

